# Influence of dielectric layer thickness and roughness on topographic effects in magnetic force microscopy

**DOI:** 10.3762/bjnano.10.106

**Published:** 2019-05-17

**Authors:** Alexander Krivcov, Jasmin Ehrler, Marc Fuhrmann, Tanja Junkers, Hildegard Möbius

**Affiliations:** 1 Department of Computer Sciences/Micro Systems Technology, University of Applied Sciences Kaiserslautern, Amerikastr. 1, 66482 Zweibrücken, Germany; 2 Polymer Reaction Design group, School of Chemistry, Monash University, Clayton VIC 3800, Australia; 3 Institute for Materials Research, Hasselt University, Martelarenlaan 42, 3500 Hasselt, Belgium

**Keywords:** capacitive coupling, electrostatic effects, magnetic force microscopy, nanoparticles, superparamagnetic iron oxide nanoparticle (SPION)

## Abstract

Magnetic force microscopy (MFM) has become a widely used tool for the characterization of magnetic properties. However, the magnetic signal can be overlapped by additional forces acting on the tip such as electrostatic forces. In this work the possibility to reduce capacitive coupling effects between tip and substrate is discussed in relation to the thickness of a dielectric layer introduced in the system. Single superparamagnetic iron oxide nanoparticles (SPIONs) are used as a model system, because their magnetic signal is contrariwise to the signal due to capacitive coupling so that it is possible to distinguish between magnetic and electric force contributions. Introducing a dielectric layer between substrate and nanoparticle the capacitive coupling can be tuned and minimized for thick layers. Using the theory of capacitive coupling and the magnetic point dipole–dipole model we could theoretically explain and experimentally prove the phase signal for single superparamagnetic nanoparticles as a function of the layer thickness of the dielectric layer. Tuning the capacitive coupling by variation of the dielectric layer thickness between nanoparticle and substrate allows the distinction between the electric and the magnetic contributions to the MFM signal. The theory also predicts decreasing topographic effects in MFM signals due to surface roughness of dielectric films with increasing film thickness.

## Introduction

MFM has become an important tool for studying magnetic properties of surface structures with submicrometer resolution [1–8]. Although the MFM signals in the so-called interleave mode are taken at a certain distance (lift height) from the sample, following the topography of the sample measured in a first scan, a total force is measured with unknown contributions from different forces. Therefore, the quantitative analysis of magnetic properties is still an issue especially because of contributions from electrostatic forces leading to topographic features in the MFM phase images [7,9–14]. Yu et al. [9] explained the topographic artifacts by electrostatic interactions. Origin of these artifacts is the work-function difference between tip and sample material. Yu et al. [10] demonstrated that topographic features can be avoided by combining MFM with electrostatic force microscopy (EFM) compensating the contact potential difference by an appropriate tip bias. Kim et al. [11] used a capacitive coupling of electrostatic force modulation to separate the magnetic from the topographic signal. In our previous paper [14], we demonstrated that Kelvin force probe microscopy (KPFM) measurements as proposed by Jaafar et al. [13] show no difference between measurements above SPIONs and measurements above the substrate. The combination of KPFM and MFM can only eliminate the electrostatic contributions for structures larger than the tip size [13]. Measuring structures with dimensions similar or smaller than the tip size KPFM does not reduce the capacitive coupling effect. During the imaging of nanoparticles a mirroring of the topography is often observed in MFM phase images [15–18]. Neves et al. [15] distinguished between magnetic and nonmagnetic nanoparticles by applying an external bias to the tip minimizing the topographical influence of the sample. Without an external tip bias a positive phase shift in the MFM image was reported for the nonmagnetic nanoparticles. Passeri et al. [19] also observed a positive phase shift for nonmagnetic niosomes in the MFM phase image. Angeloni et al. [16] discussed the topography-induced positive phase shift for small SPIONs aggregates by capacitive coupling effects. In order to distinguish electrostatic and magnetic forces Angeloni et al. [18] employed a controlled change of the tip magnetization. They demonstrated this new method by measuring superparamagnetic nanoparticles but also discussed current limitations of this technique such as artifacts in the magnetic image due to instrumental parameters as well as incomplete demagnetization of the probe. In our previous work, we theoretically explained and experimentally proved that the positive phase shift above nanoparticles derives from capacitive coupling between tip and substrate [14]. The increase of the tip–substrate distance in the interleave mode above the nanoparticle leads to a reduction of the electrostatic forces resulting in a positive phase shift. Furthermore methods to reduce the capacitive coupling were discussed, e.g., measurements on substrates with a minimized work-function difference between tip and substrate or the usage of a tip with smaller radius. In [20] we demonstrated that capacitive coupling effects vanish investigating nanoparticles embedded in a polymer matrix.

In this work we show that capacitive coupling effects can be reduced by using a dielectric layer between substrate and nanoparticles. Magnetic nanoparticles in the superparamagnetic state are used in this work in order to distinguish between electrostatic and magnetic signal. The magnetic vector of the superparamagnetic nanoparticle is aligned along the field of the probe resulting in an attractive force. In contrast, the electrostatic force due to topography changes is a repulsive force.

Simulations as well as experiments show that an increase of the dielectric layer thickness between nanoparticle and substrate leads to a decrease of the capacitive coupling.

The theoretical model described in this paper also predicts decreasing topographic effects in MFM signals due to surface roughness of dielectric films with increasing film thickness.

## Theory

### Capacitive coupling effects in MFM on nanoparticles

**Figure 1:**
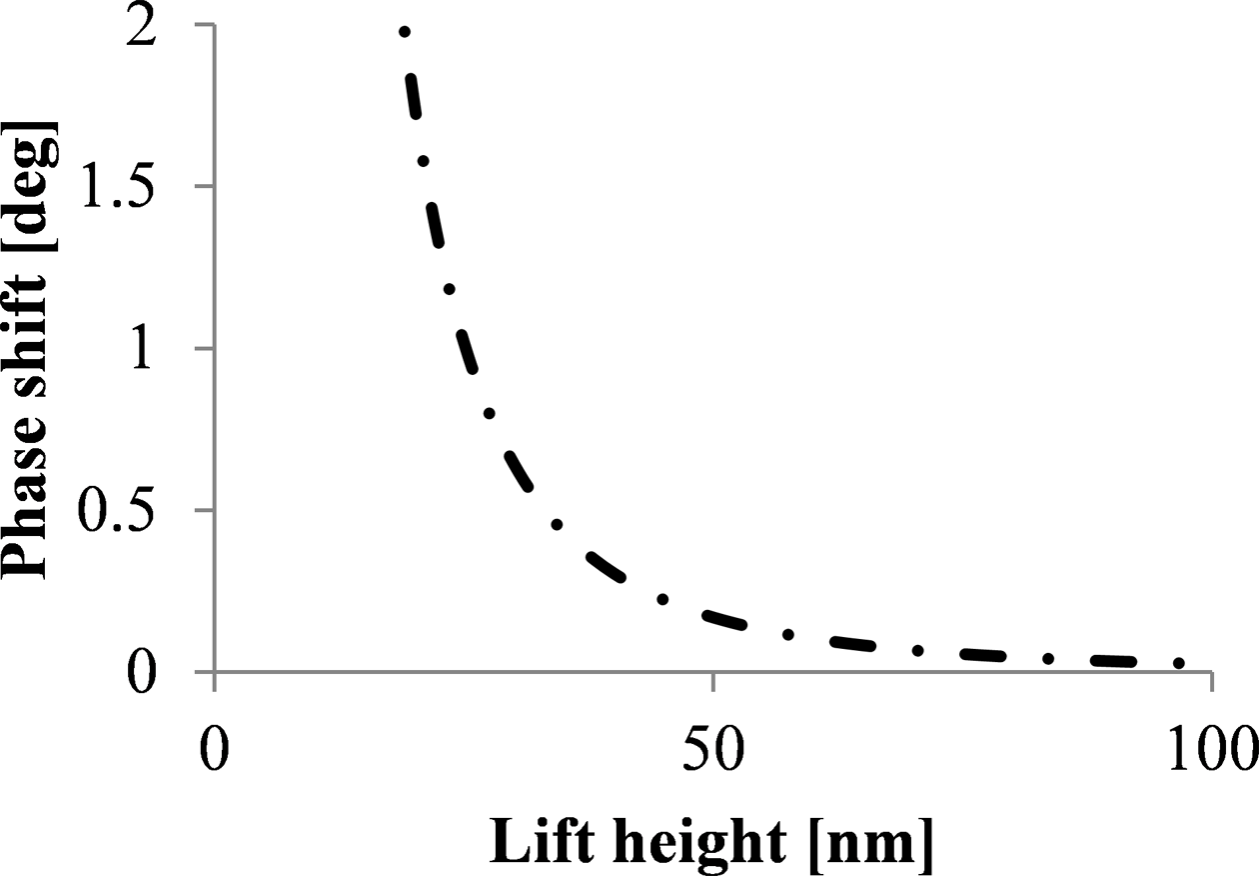
Phase shift as a function of the tip–substrate distance *z*; calculated for a silicon substrate with *V*_CPD_ = 0.35 V, a tip radius of 84 nm and a nanoparticle diameter of 10 nm on a flat substrate.

In our previous work we proposed a theory of a capacitive coupling between tip and substrate explaining the mirroring of the topography in MFM phase images, and thus a positive phase shift, when measuring nanoparticles [14]. While measuring in interleave mode the distance *z* between the probe and the substrate increases above the nanoparticles (*z + d*, with *d* nanoparticle diameter) resulting in a positive phase shift:

[1] $$\Delta \varphi_{\text{el}}=-\frac{Q}{k}\varepsilon_{0}\left[\frac{A}{{\left(z+d\right)}^{3}}{\left(V_{\text{CPD}}\right)}^{2}-\frac{A}{z^{3}}{\left(V_{\text{CPD}}\right)}^{2}\right],$$

with *A* being the effective capacitive area, *z* the lift height, *d* the nanoparticle diameter, *V*_CPD_ the contact potential difference between tip and substrate, *Q* the quality factor, *k* the spring constant of the cantilever, and ε_0_ the dielectric constant of vacuum. The effective area of the capacitor is calculated taking the curvature of the tip into account [14]. 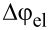 represents the difference in phase shift above and beside the nanoparticle. As the effective interaction area of the tip and the single nanoparticle is less than 2% of the interaction area between tip and substrate the contribution of the capacitance between tip and SPION and the self-capacitance of the SPION can be neglected [14]. The capacitive coupling significantly increases the phase shift for small lift heights as shown in Figure 1.

The capacitive coupling is decreasing with increasing lift height (Figure 1). However, in order to measure the magnetic signal of single SPIONs, the distance between nanoparticle and tip has to be in the range of 10 to 30 nm in order to get a magnetic interaction above the detection limit [14]. At this range the attractive magnetic interactions of SPIONs are often hidden by repulsive electrostatic interactions due to the changes in capacitive coupling. In [14] possibilities are discussed to minimize the capacitive coupling, e.g., by using a substrate with minimized work-function difference between tip and substrate or by using a sharper tip.

**Figure 2:**
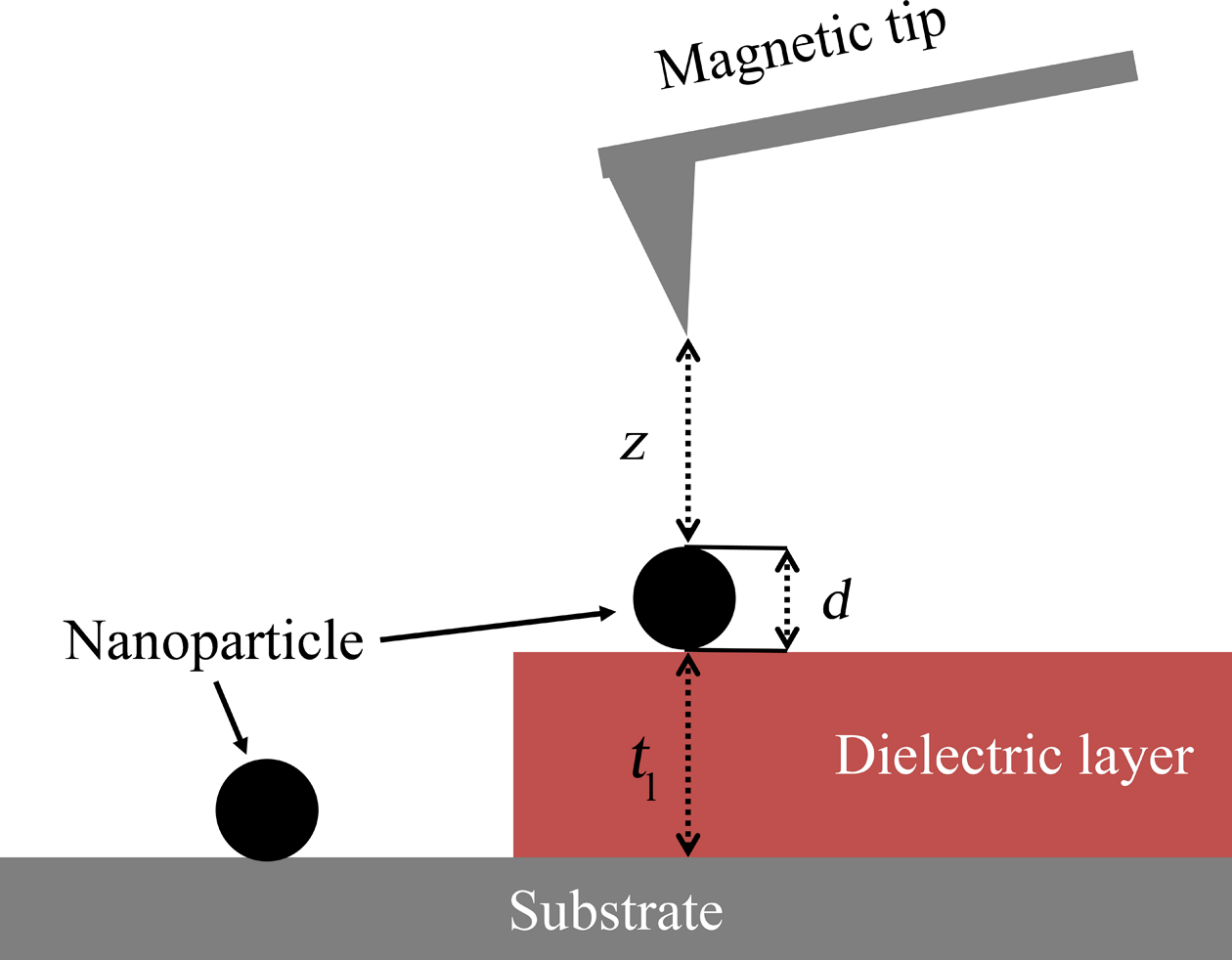
Substrate without dielectric layer and with dielectric layer. The dielectric layer allows the tip to get closer to the nanoparticle without disturbing capacitive coupling due to interaction of tip and substrate.

Another possibility to minimize the capacitive coupling is the use of a dielectric layer between substrate and nanoparticle, as shown in Figure 2. This results in a reduction of capacitive coupling due to the larger distance between tip and substrate even for small lift heights above the nanoparticles assuring a strong magnetic interaction between nanoparticle and tip.

Taking into account a dielectric layer the formula for capacitive coupling can be modified as follows:

[2] $$\Delta \varphi_{\text{el}}=-\frac{Q}{k}\varepsilon_{0}\left[\frac{A}{{\left(z+d+t_{\text{eff}}\right)}^{3}}{\left(V_{\text{CPD}}\right)}^{2}-\frac{A}{{\left(z+t_{\text{eff}}\right)}^{3}}{\left(V_{\text{CPD}}\right)}^{2}\right].$$

The effective film thickness *t*_eff_ of the dielectric layer is included in the distance between tip and substrate to calculate the capacitive coupling of the tip with the substrate beneath the dielectric layer. The effective thickness is calculated as follows:

[3] $$t_{\text{eff}}=\frac{t_{\text{l}}}{\varepsilon_{\text{r}}}.$$

ε_R_ represents the dielectric constant and *t*_l_ the real film thickness of the dielectric layer. The dielectric constant for the layer used in this work is 3.1 [21].

**Figure 3:**
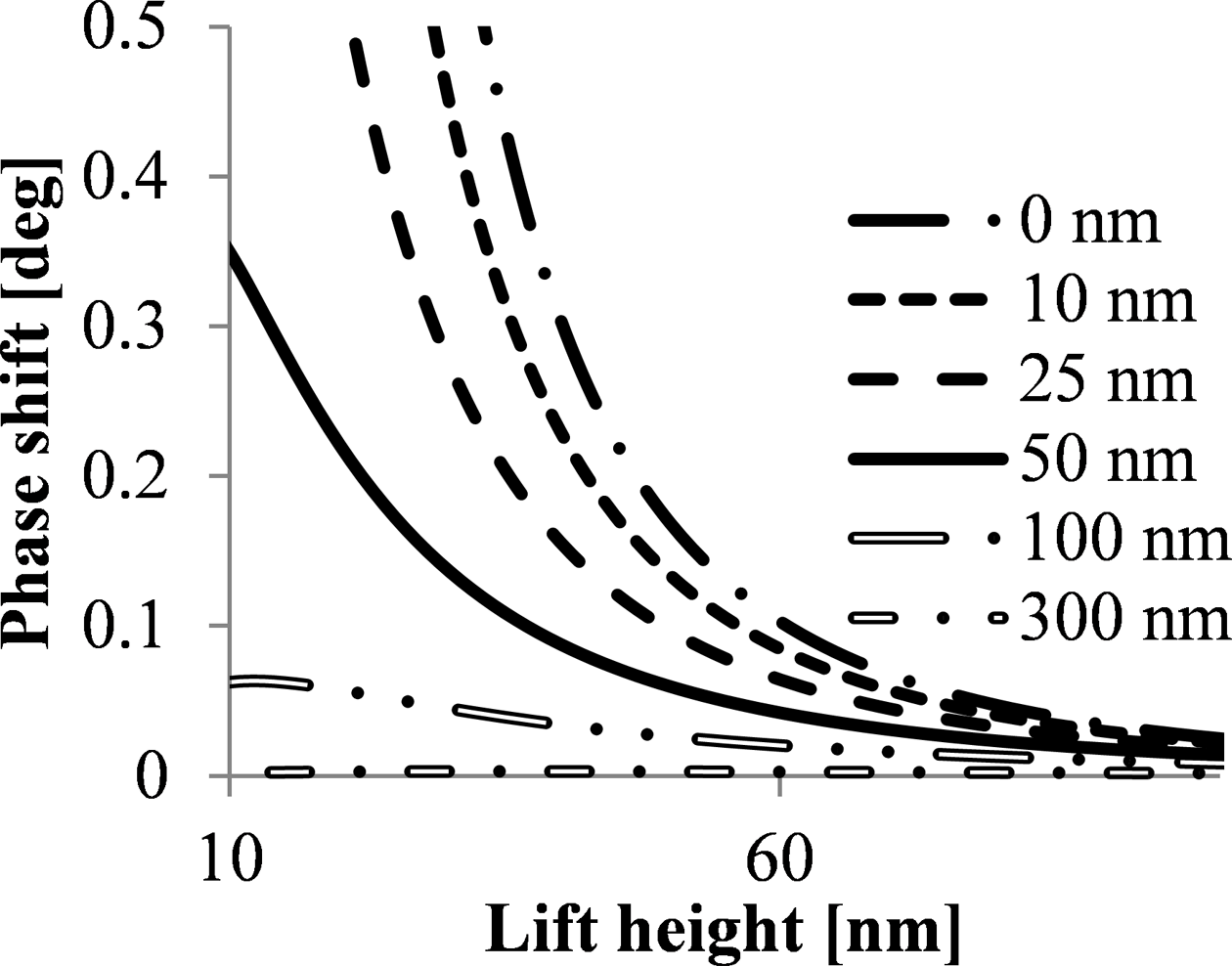
Phase shift due to capacitive coupling as a function of the lift height for nanoparticles with 10 nm diameter on a silicon substrate with dielectric layer. The thickness of dielectric layer is 0 nm (silicon surface), 10 nm, 20 nm, 25 nm and 50 nm; calculated for a tip radius of 84 nm, *V*_CPD_ = 0.35 V and a dielectric constant of 3.1.

Figure 3 compares the phase shift due to capacitive coupling of a nanoparticle lying on a flat substrate with the phase shift for a nanoparticle lying on dielectric layers with different layer thicknesses. All simulations were carried out assuming the absence of trapped charges on the dielectric layer.

Introducing a dielectric layer in the system the capacitive coupling effect is significantly reduced for lift heights below 40 nm. It can therefore be concluded that dielectric layer thicknesses larger than 100 nm allow the detection of weak magnetic signals with reduced overlaying electrostatic effects.

### Capacitive coupling effects in MFM on rough surfaces

**Figure 4:**
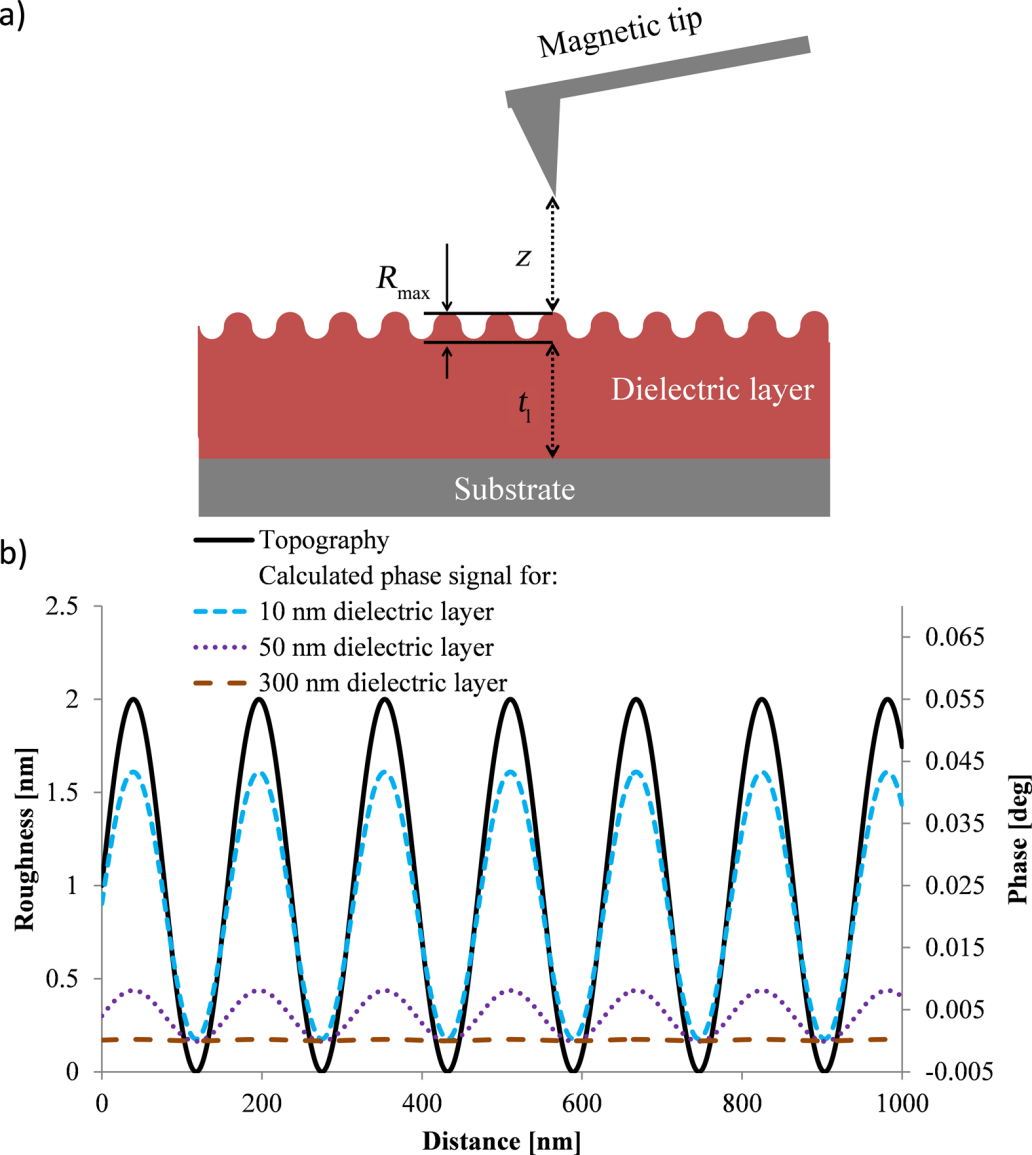
a) Sketch of a MFM measurement of a dielectric layer with defined roughness (*R*_max_); b) Simulation for rough surfaces using a sine wave depicting the roughness and assuming a peak to peak distance, *R_max_* in Figure 4a, of 2 nm; calculated for a silicon substrate with *V*_CPD_ of 0.35 V, tip radius of 84 nm, 20 nm lift height and dielectric constant of the dielectric layer of 3.1.

The considerations about capacitive coupling effects on nanoparticles can be generalized assuming rough surfaces depicted in Figure 4a.

The distance changes between tip and substrate in the interleave mode due to the surface roughness (measured in the first scan) also lead to capacitive coupling effects and positive phase shifts in the MFM image as shown in Figure 4b. For dielectric layer with a thickness on the silicon substrate in the range of 10 nm the capacitive coupling due to the roughness leads to topographical mirroring in the MFM signal whereas the roughness of layers of several 100 nm thickness has no influence on the MFM phase signal. Although the idealized calculations neglect trapped charges on the dielectric layer the simulations reveal a significant reduction of capacitive coupling using a dielectric layer.

### Magnetic forces between tip and SPION

Single SPIONs are chosen as a model system to investigate the superposition of magnetic and electrostatic contributions in the MFM phase shift. Theoretical estimates based on vibrating sample magnetometer (VSM) measurements (Figure S2, Supporting Information File 1) reveal that the magnetic field of the probe with a magnetic moment of 3·10^−16^ A·m^2^ is sufficient to induce a magnetic moment at lift heights up to 150 nm in superparamagnetic nanoparticles with 10 nm diameter. This results in attractive forces and, thus, negative phase shifts in MFM measurements. Therefore the magnetic signal is contrariwise to the signal of capacitive coupling described above.

**Figure 5:**
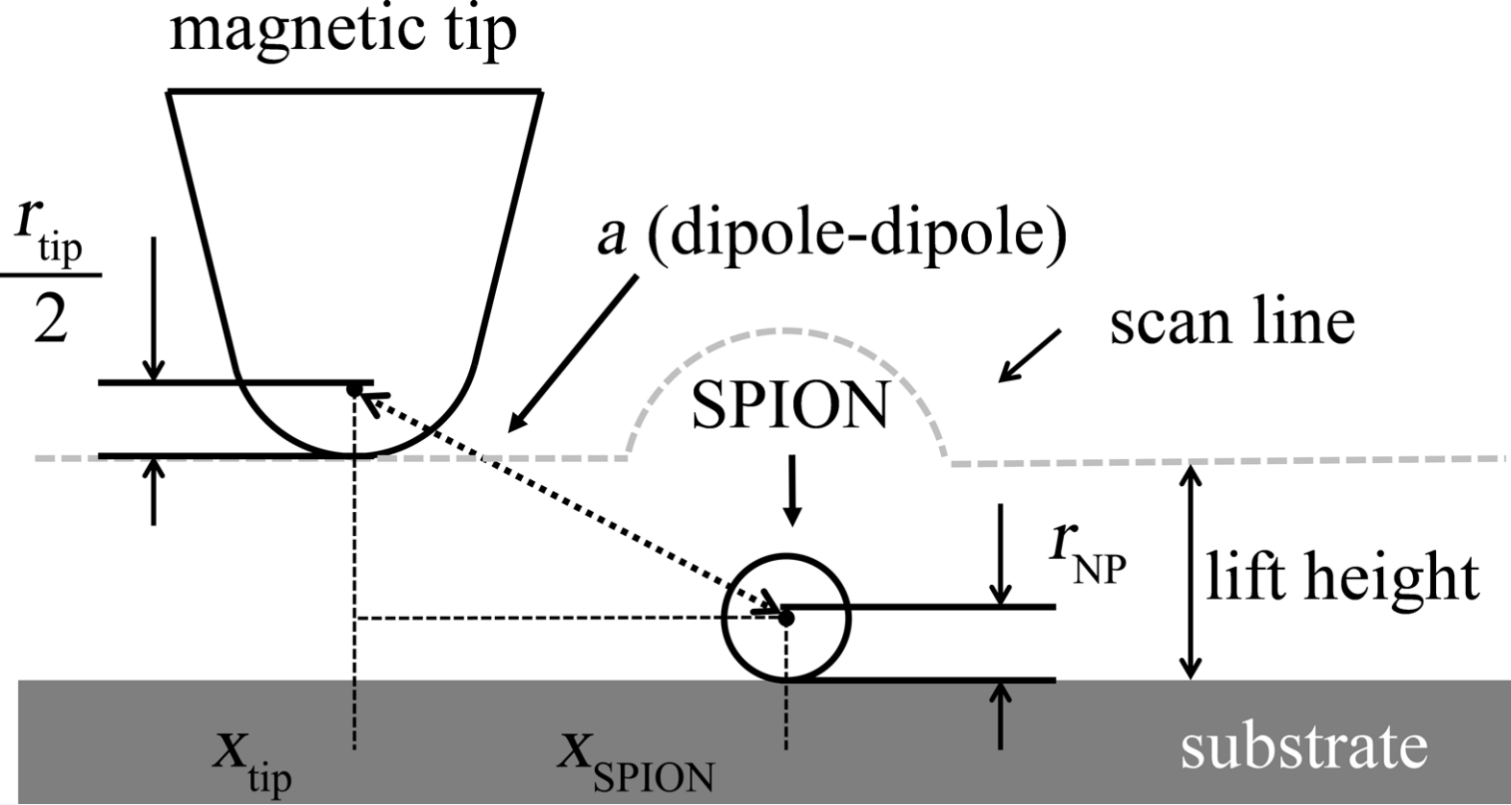
Schematic representation of the distance between tip and nanoparticle dipole during the interleave measurements.

The magnetic point dipole**–**dipole approximation is used to calculate the magnetic force gradient acting on the tip due to the interaction between a spherical superparamagnetic nanoparticle and the tip, approximated by a uniform magnetized sphere [20,22–23]:

[4] $$\Delta \varphi_{\text{mag}}=-\frac{Q}{k}\frac{\partial F}{\partial z}=-\frac{Q}{k}\frac{6\mu_{0}m_{\text{p}}m_{\text{tip}}}{\pi {\left(a\right)}^{5}},$$

where *Q* is the quality factor of the cantilever, *k* is the spring constant, µ_0_ is the vacuum permeability, *m*_p_ is the magnetic moment of the nanoparticle, *m*_tip_ is the magnetic moment of the tip, and *a* is the distance between the two dipoles and is shown schematically in Figure 5. The position of the tip dipole is assumed to be at the half radius of the tip [17].

### Cross section simulation of MFM phase

**Figure 6:**
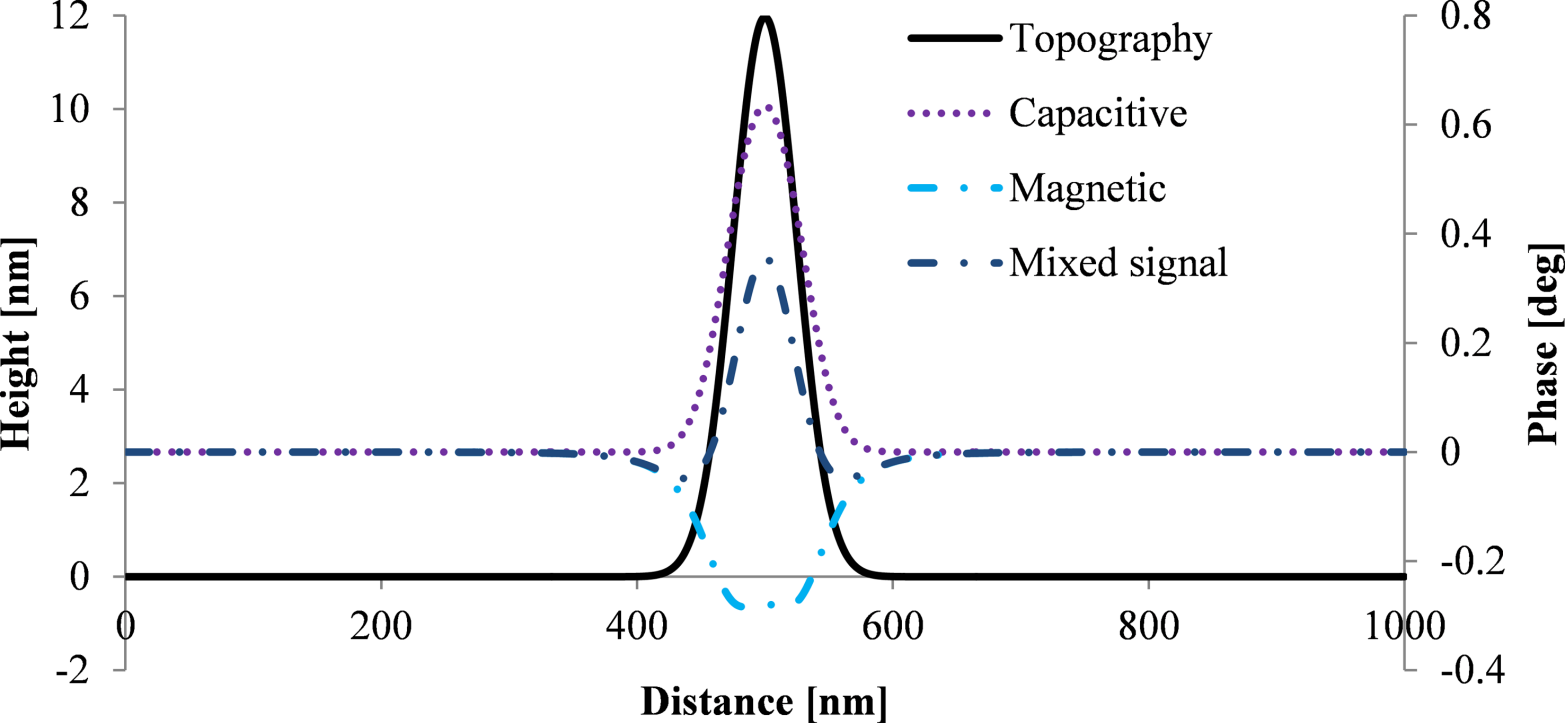
Simulation of the MFM phase for a single SPION using a Gaussian topographic profile corresponding to a SPION with a diameter of 12 nm and a lift height of 20 nm. *V*_CPD_ for the electrostatic force is 0.35 V. A tip volume magnetization of 4.5·10^−15^ A·m^2^ and a specific magnetization of the SPION of 80 A·m^2^·kg^−1^ were used for calculations of the magnetic force.

The first scan of MFM measurements provides a topographic image displaying a convolution of the tip and the nanoparticle [24]. The topographic cross section is simulated by using a Gaussian profile at the position of the nanoparticle (black line in Figure 6). The width of the Gaussian profile is much broader than the width of the nanoparticle (assumed to be 12 nm) because of the convolution with the tip with a radius of 84 nm. Both magnetic and electric forces for a single SPION can be calculated as a function of the horizontal position of the tip corresponding to a cross section of the MFM image. The vertical distance changes in the second scan (interleave scan with a certain lift height following the topography of the first scan) lead to a positive phase shift due to capacitive coupling and can be calculated by Equation 1 for each horizontal position of the tip. In order to determine the negative phase shift due to the magnetic interaction between tip and nanoparticle according to Equation 4, the distance *a* between the point dipole representing the tip and the point dipole representing the nanoparticle can be calculated for each horizontal position of the tip as follows:

[5] $$a=\sqrt{{\left[\left(h+z+\frac{r_{\text{tip}}}{2}\right)-r_{\text{SPION}}\right]}^{2}+{\left(x_{\text{tip}}-x_{\text{SPION}}\right)}^{2}},$$

where *a* is the distance between the dipoles, *h* is the height of the topography and *z* is the lift height. Figure 6 shows a simulation of both forces, capacitive (dotted purple line in Figure 6) and magnetic (dashed light blue line in Figure 6), for a single nanoparticle with 12 nm diameter on a silicon substrate. The dashed-dotted dark blue line presents the overall signal comprising electrostatic and magnetic interaction.

Electrostatic forces are stronger than the magnetic forces resulting in a positive phase shift above the nanoparticle. However, weak attractions are expected in a ring around the nanoparticles because the magnetic signal is broader than the topographical signal (the half width of the magnetic signal is ca. 100 nm and that of the electric signal is ca. 60 nm in this simulation).

When a dielectric layer is introduced the capacitive coupling can be reduced so that the overall phase signal becomes negative. Thus, tuning the phase shift due to capacitive coupling by introducing a dielectric layer suppresses the electrostatic interaction and allows the visualization of magnetic contributions to the MFM phase signal.

## Results and Discussion

### Minimization of capacitive coupling through dielectric layer

**Figure 7:**
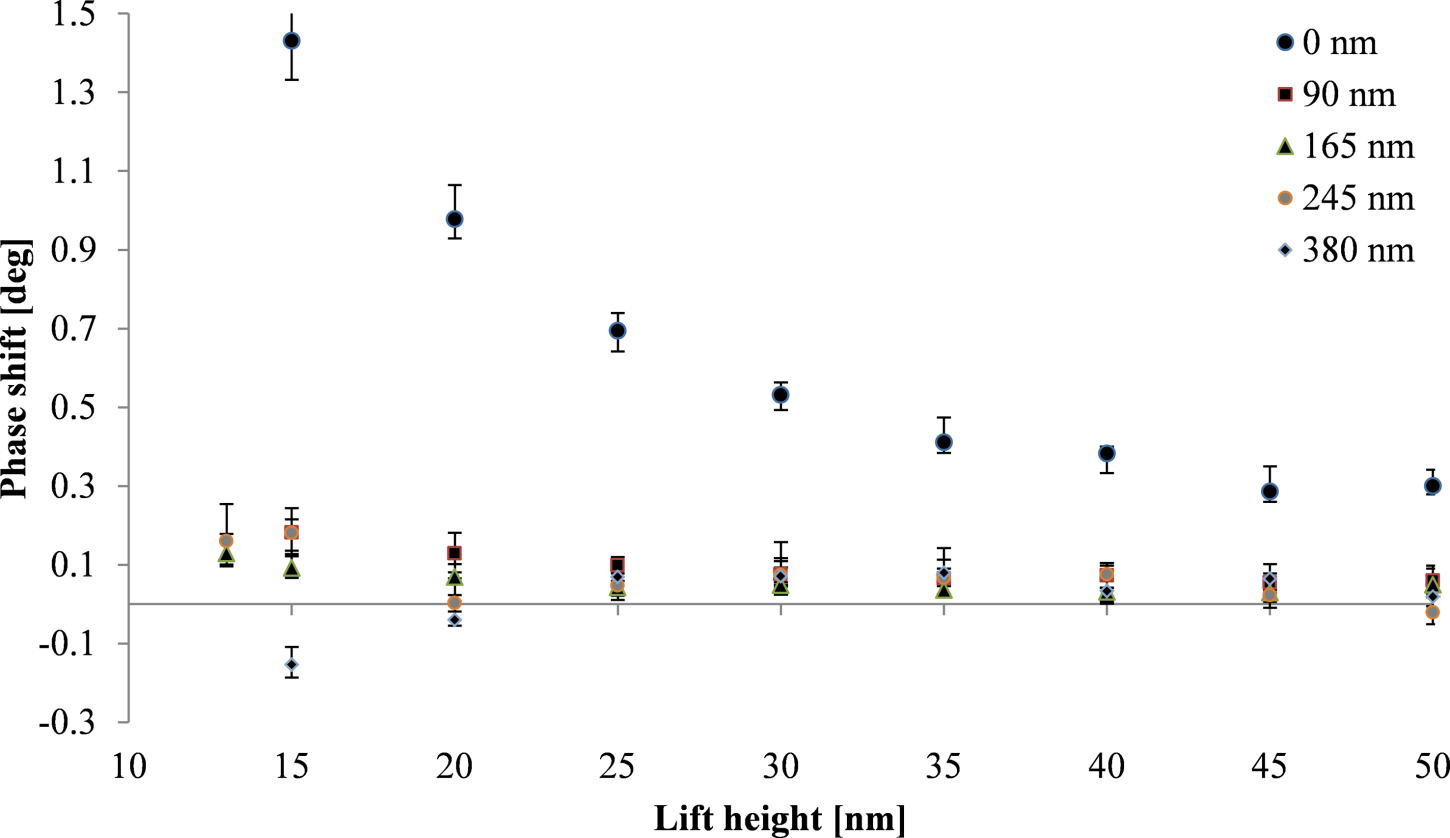
Phase shift above nanoparticles (10 ± 2 nm) on dielectric layers of various thicknesses as a function of the lift height. The measurements were carried out using a tip with high magnetic moment (ASYMFM-HM) to assure magnetic sensitivity.

According to theory the capacitive coupling can be reduced by increasing the distance between tip and substrate. This can be achieved by adding a dielectric layer between substrate surface and SPION. Figure 7 shows the phase shift as a function of lift height for substrates with different dielectric layer thicknesses ranging from 0 nm (no layer) up to 380 nm. The measurements are carried out with a tip with high magnetic moment (ASYMFM-HM tip) in order to obtain a strong magnetic interaction between tip and SPIONs. In case of SPIONs lying directly on the silicon substrate the capacitive coupling is dominant and completely hides the magnetic signal. Adding a dielectric layer between silicon substrate and the nanoparticle, the capacitive coupling is reduced. The capacitive coupling is significantly decreasing with increasing layer thickness of the dielectric layer. For a dielectric layer with 380 nm layer thickness the magnetic interaction is dominating the phase signal for small lift heights resulting in attraction and, therefore, negative phase shift for lift heights of 15 and 20 nm.

**Figure 8:**
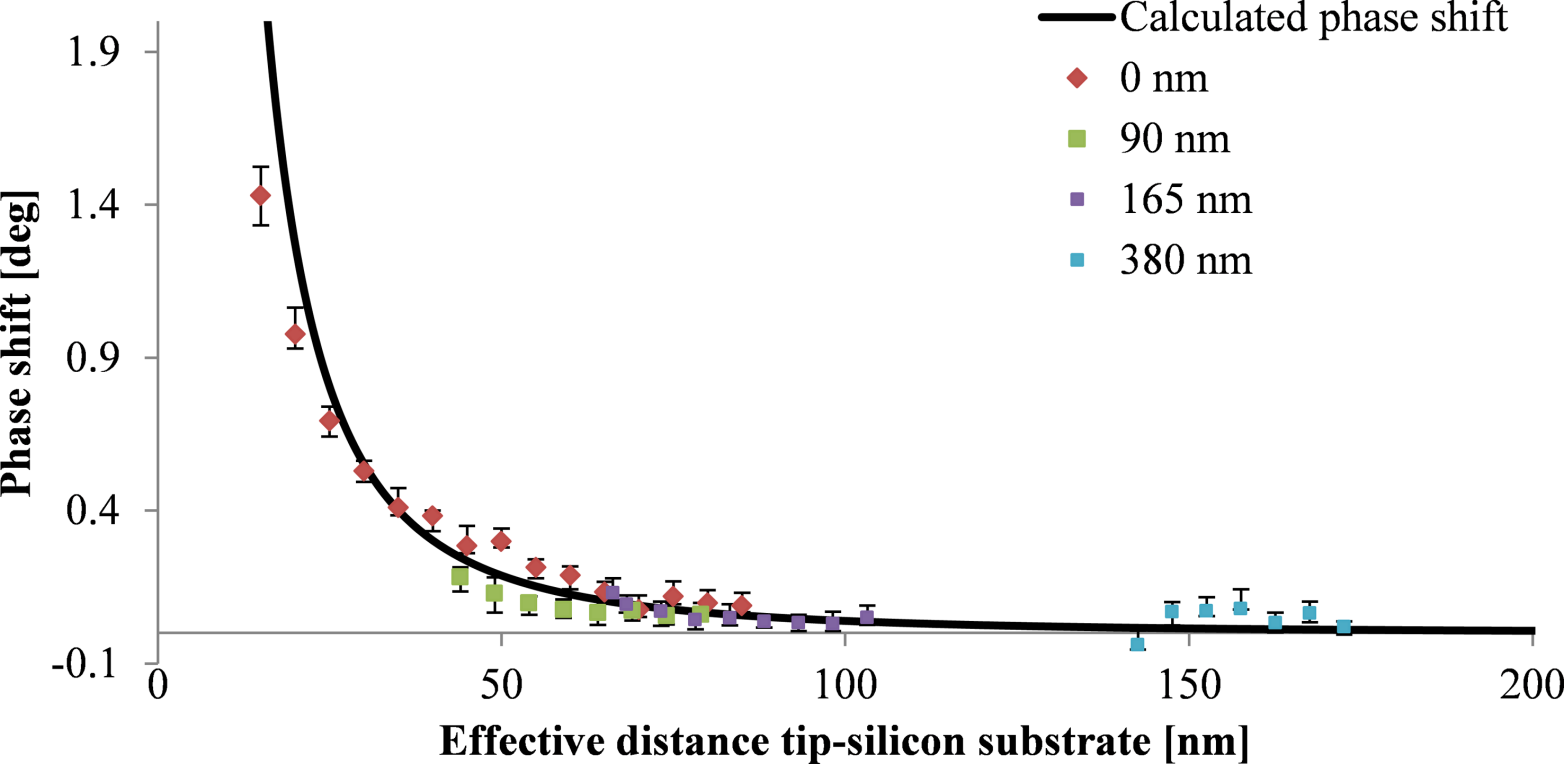
Calculated (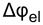; black line) and measured phase shift for single nanoparticles with 10 ± 2 nm diameter on silicon substrates with dielectric layers of different thicknesses of 0, 90, 165 and 380 nm; calculated for a silicon substrate with *V*_CPD_ of 0.35 V, and a tip radius of 84 nm taking into account the dielectric constant of 3.1 of the dielectric layer. The effective distance is determined by lift height *z*, particle diameter *d* and *t*_eff_.

Figure 8 shows the phase shift as a function of the effective tip–substrate distance, (*z* + *t*_eff_, Figure 2) for samples with layer thicknesses varying from 0 to 380 nm. For each layer thickness the phase shift above the nanoparticle has been measured as a function of the lift height *z* (Figure 2). The black line shows the theoretical phase shift for capacitive coupling with a silicon substrate indicating the decrease of the capacitive coupling with increasing dielectric layer thickness as discussed above.

**Figure 9:**
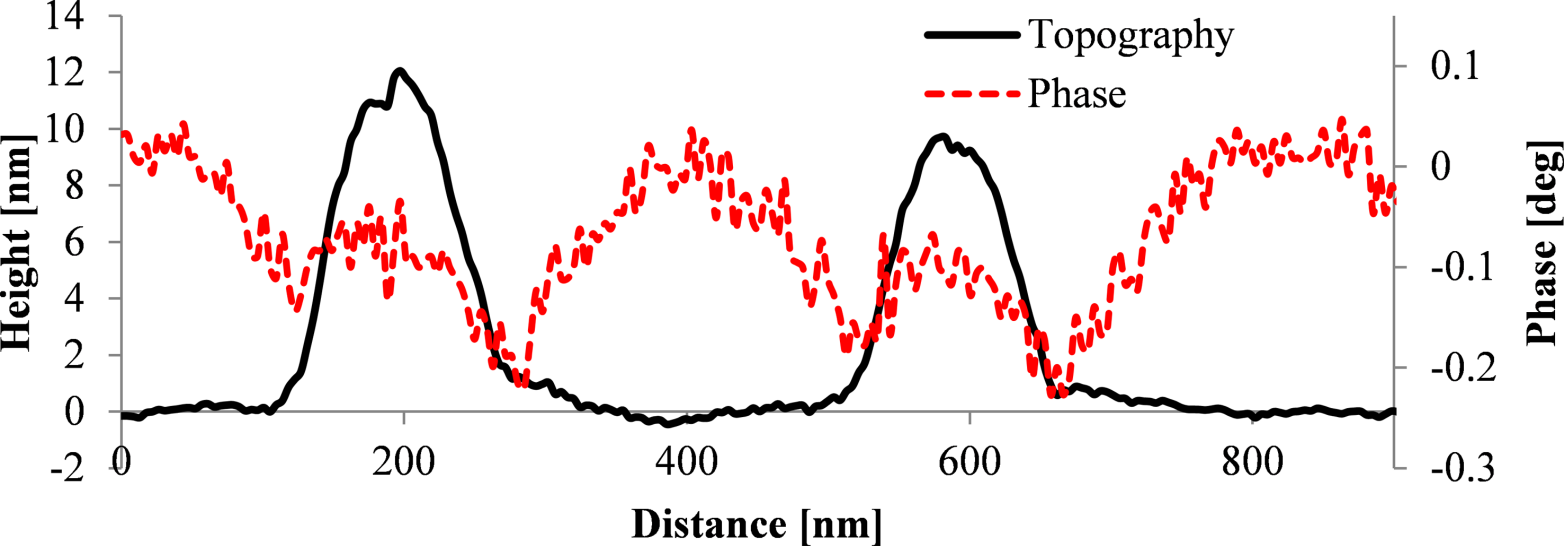
Measurement of a single SPION with 10 ± 2 nm diameter on a silicon substrate with a dielectric layer (380 nm layer thickness) recorded with an ASYMFM-HM tip at a lift height of 20 nm.

We observe a significant decrease of the capacitive coupling between tip and substrate with increasing layer thickness. The positive phase shift above the nanoparticles can be reduced by a factor of about seven introducing dielectric layers with thicknesses of 90 nm and more. Trapped charges on the surface of the dielectric layer might limit the minimization of the capacitive coupling and result in a weak capacitive coupling between tip and dielectric layer. Figure 9 shows magnetic nanoparticles on a silicon substrate with a spin-coated dielectric layer of 380 nm thickness. The repulsive force of capacitive coupling is reduced significantly. However, an increase of the phase shift can still be observed directly above the nanoparticles indicating a remaining weak capacitive coupling.

**Figure 10:**
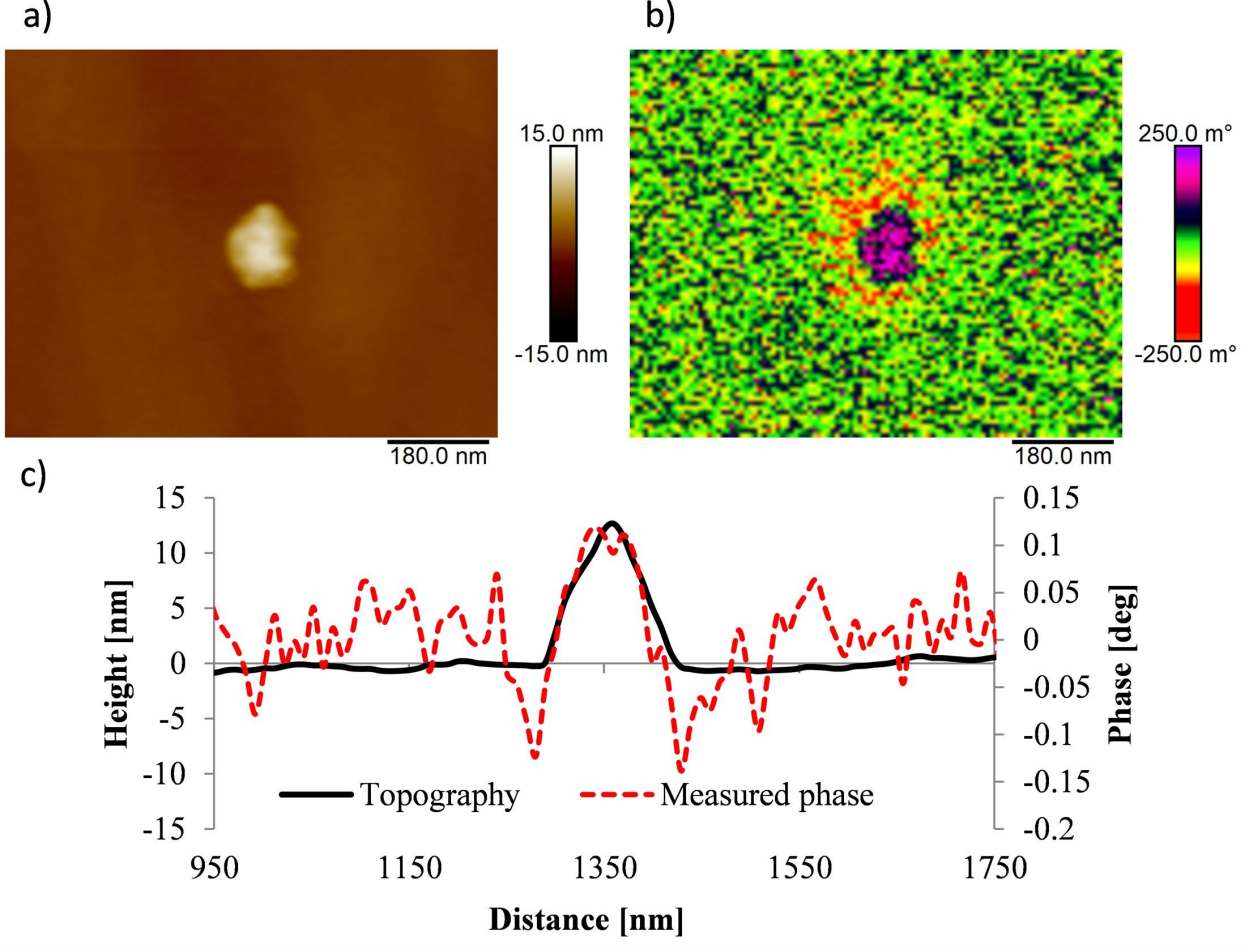
Measurement of a single SPION with 12 ± 1 nm diameter on a silicon substrate recorded with an ASYMFM-HM tip. a) Topography image, b) phase image at 20 nm lift height and c) cross section of topography image (black line) and of phase image (red line).

Figure 10 shows a cross section of the topography image as well as of the phase image for a single SPION on a silicon substrate. For the single SPION lying directly on the silicon surface a strong repulsion indicated by a positive phase shift is measured for lift heights of 50 nm or less. Additionally, a negative phase shift is observed around the nanoparticle indicating an attractive magnetic force. This magnetic aureole around the nanoparticle is due to the broader magnetic signal compared to the electrostatic signal from capacitive coupling. This behavior is in accordance with our simulations combining magnetic and electrostatic interactions resulting in a ring of negative phase shift (Figure 6).

Hence, MFM measurements on single SPIONs reveal contributions of electrostatic forces in form of a positive phase shift above the nanoparticles as well as contributions of magnetic forces observed as a ring, a magnetic aureole, of negative phase shift around the nanoparticle.

## Conclusion

In summary we could explain the specific MFM phase characteristic often seen in measurements of SPIONs. The phase image arising due to interplay of electrostatic and magnetic forces could be explained. We showed that capacitive coupling effects can be reduced by including a dielectric layer between substrate and nanoparticle. The capacitive coupling decreases with increasing layer thickness since the distance between tip and substrate is increased. The theoretical model described in this paper also predicts decreasing topographic effects in MFM signals due to surface roughness of dielectric films with increasing film thickness.

## Experimental

The MFM measurements in this work were carried out under ambient conditions using a Bruker Dimension Icon atomic force microscope. The topography of the samples was measured in tapping mode and the phase images in interleave mode at a certain lift height. The changes in amplitude indicate the topography changes in tapping mode. The amplitude of the tip oscillation is 50 nm in order to increase the signal-to-noise ratio. The resolution of the images is 254 measuring points per line, and the scan speed is 0.9 Hz. The force gradient is detected by phase shifts in interleave mode. A magnetic ASYMFM-HM tip was used for all measurements in order to assure a high magnetic sensitivity. The ASYMFM-HM tip has a resonance frequency of 75 kHz, a radius of 84 nm and a magnetic moment of 3·10^−16^ A·m^2^ due to the magnetic CoCr coating. The MFM measurements were processed using the NanoScope analysis software. A flatten command of 1st order was used on topography and phase images shown in this paper to remove offset and slope of the measured data.

The SPIONs with 10 ± 2 nm diameter with oleic acid as stabilizing ligand were used as received (Merck). A single-crystal silicon substrate with <100> orientation (Siegert Wafer) was cut in 2 cm^2^ squares. The dielectric layers were spin-coated on silicon substrates at 3000 rpm using a closed-system spin-coater with rotating lid (BLE). Bisphenol A-epichlorohydrin resin was used as dielectric layer. The resin is the hard component of AR-P 5910 resist (Allresist) with a dielectric constant of 3.1 [21]. The thickness was varied by different dilutions of the resist using the AR 300-12 thinner (Allresist). The layer thickness and the degree of dilutions are shown in Table S1 (Supporting Information File 1). The thickness of the layers was measured using the AFM tip-scratch method.

## Supporting Information

File 1 Additional experimental details.

## References

[1] Kim D, Chung N-K, Allen S, Tendler S J B, Park J W (2012). ACS Nano.

[2] Schreiber S, Savla M, Pelekhov D V, Iscru D F, Selcu C, Hammel P C, Agarwal G (2008). Small.

[3] Körnig A, Hartmann M A, Teichert C, Fratzl P, Faivre D (2014). J Phys D: Appl Phys.

[4] Moya C, Iglesias-Freire Ó, Batlle X, Labarta A, Asenjo A (2015). Nanoscale.

[5] Torre B, Bertoni G, Fragouli D, Falqui A, Salerno M, Diaspro A, Cingolani R, Athanassiou A (2011). Sci Rep.

[6] Sievers S, Braun K-F, Eberbeck D, Gustafsson S, Olsson E, Schumacher H W, Siegner U (2012). Small.

[7] Li J-R, Lewandowski B R, Xu S, Garno J C (2009). Anal Chem (Washington, DC, U S).

[8] Li X, Lu W, Song Y, Wang Y, Chen A, Yan B, Yoshimura S, Saito H (2016). Sci Rep.

[9] Yu J, Ahner J, Weller D (2004). J Appl Phys.

[10] Yu J, Ahner J, Weller D (2003). Appl Phys Lett.

[11] Kim B I (2009). Rev Sci Instrum.

[12] Schwarz A, Wiesendanger R (2008). Nano Today.

[13] Jaafar M, Iglesias-Freire O, Serrano-Ramón L, Ibarra M R, de Teresa J M, Asenjo A (2011). Beilstein J Nanotechnol.

[14] Krivcov A, Junkers T, Möbius H (2018). J Phys Commun.

[15] Neves C S, Quaresma P, Baptista P V, Carvalho P A, Araújo J P, Pereira E, Eaton P (2010). Nanotechnology.

[16] Angeloni L, Passeri D, Reggente M, Rossi M, Mantovani D, Lazzaro L, Nepi F, De Angelis F, Barteri M (2015). AIP Conf Proc.

[17] Raşa M, Kuipers B W M, Philipse A P (2002). J Colloid Interface Sci.

[18] Angeloni L, Passeri D, Reggente M, Mantovani D, Rossi M (2016). Sci Rep.

[19] Passeri D, Dong C, Reggente M, Angeloni L, Barteri M, Scaramuzzo F A, De Angelis F, Marinelli F, Antonelli F, Rinaldi F (2014). Biomatter.

[20] Krivcov A, Schneider J, Junkers T, Möbius H (2018). Phys Status Solidi A.

[21] (2019). Technical Data Sheet for AR-P 3100 Resist by Allresist.

[22] Hartmann U (1989). Phys Lett A.

[23] Edwards B F, Riffe D M, Ji J-Y, Booth W A (2017). Am J Phys.

[24] Serem W K, Lusker K L, Garno J C (2010). Using Scanning Probe Microscopy to Characterize Nanoparticles and Nanocrystals. Encyclopedia of Analytical Chemistry.

