# Supplementary material for: Influence of dielectric layer thickness and roughness on topographic effects in magnetic force microscopy
Source: Beilstein J Nanotechnol. 2019 May 17;10:1056–64. doi: 10.3762/bjnano.10.106 (PMC6541333; doi:10.3762/bjnano.10.106)
Supplement: File 1 — Additional experimental details. [file Beilstein_J_Nanotechnol-10-1056-s001.pdf]

BEILSTEIN JOURNAL OF NANOTECHNOLOGY

Supporting Information

for

Influence of dielectric layer thickness and roughness on topographic effects in magnetic force microscopy

Alexander Krivcov, Jasmin Ehrler, Marc Fuhrmann, Tanja Junkers and Hildegard Möbius

Beilstein J. Nanotechnol. **2019,** *10,* 1056–1064. doi:10.3762/bjnano.10.106

Additional experimental details

License and Terms: This is a supporting information file under the terms of the Creative Commons Attribution License (http://creativecommons.org/licenses/by/4.0). Please note that the reuse, redistribution and reproduction in particular requires that the authors and source are credited.
The license is subject to the *Beilstein Journal of Nanotechnology* terms and conditions: (https://www.beilstein-journals.org/bjnano)

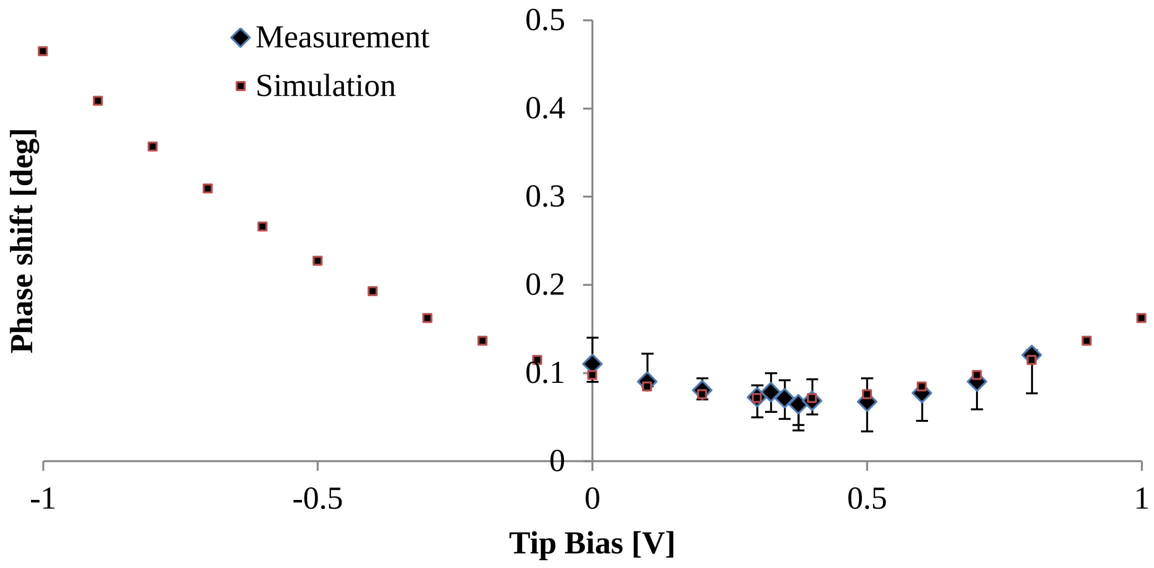

Figure S1: Measured and simulated EFM curve for silicon substrate with ASYMFM-HM tip taken at 20 nm lift height. The contact potential difference between the tip and the substrate is determined to be 0.35 ± 0.05 V.

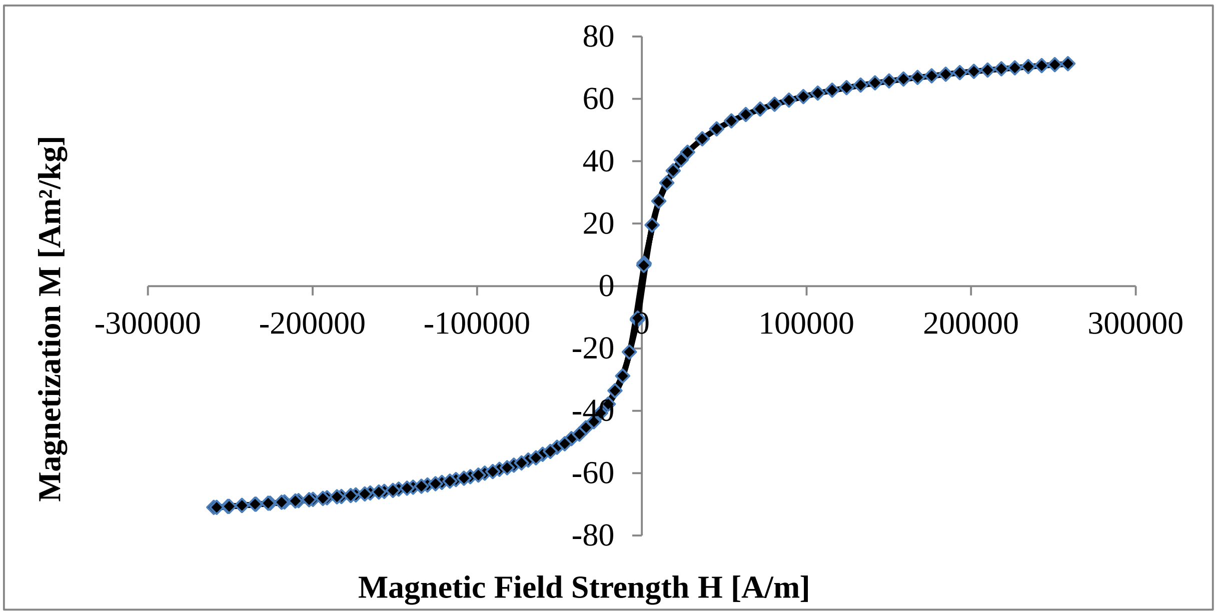

Figure S2: Vibrating sample magnetometer (VSM) measurement of superparamagnetic iron oxide nanoparticles (SPIONs) with 10 nm diameter.

Table S1: Layer thickness for resist with different degree of dilutions at 3000 rpm rotation speed.

dilution<br>resist/thinner	layer thickness<br>[nm]
1:1	380 ± 12
6:7	300 ± 10
5:7	245 ± 8
4:7	200 ± 5
3:7	165 ± 3
2:7	90 ± 2
1:7	25 ± 1
